# What prevents *Struthio camelus* and *Dromaius novaehollandiae* (Palaeognathae) from choking? A novel anatomical mechanism in ratites, the linguo-laryngeal apparatus

**DOI:** 10.1186/1742-9994-9-11

**Published:** 2012-05-31

**Authors:** Martina R Crole, John T Soley

**Affiliations:** 1 Department of Anatomy and Physiology, Faculty of Veterinary Science, University of Pretoria, Private Bag X04, Onderstepoort, 0110, South Africa

**Keywords:** *Struthio camelus*, *Dromaius novaehollandiae*, Glottis, Swallowing, Protection, Linguo-laryngeal apparatus

## Abstract

**Background:**

The avian glottis channels air from the oropharynx to the trachea and is situated on an elevated structure, the laryngeal mound. It is imperative that the glottis be protected and closed during swallowing, which in mammals is achieved by covering the glottis with the epiglottis, as well as by adduction of the arytenoid cartilages. An epiglottis, however, is reportedly absent in birds. Ratites such as *Struthio camelus* and *Dromaius novaehollandiae* possess a very wide glottis in comparison to other birds. The question therefore arises as to how these large birds avoid inhalation of ingesta through a wide glottis, with apparently little protection, particularly as their feeding method involves throwing the food over the glottis to land in the proximal esophagus.

**Results:**

In *S. camelus* when the glottis was closed and the tongue body retracted, the smooth tongue root became highly folded and the rostral portion of the laryngeal mound was encased by the pocket in the base of the ∩ − shaped tongue body. In this position the lingual papillae also hooked over the most rostral laryngeal projections. However, in *D. novaehollandiae*, retraction of the tongue body over the closed glottis resulted in the prominent, triangular tongue root sliding over the rostral portion of the laryngeal mound. In both *S. camelus* and *D. novaehollandiae* these actions resulted in the rostral portion of the laryngeal mound and weakest point of the adducted glottis being enclosed and stabilised.

**Conclusions:**

Only after conducting a comparative study between these two birds using fresh specimens did it become clear how specific morphological peculiarities were perfectly specialised to assist in the closure and protection of the wide glottis. We identify, describe and propose a unique anatomical mechanism in ratites, which may functionally replace an epiglottis; the linguo-laryngeal apparatus.

## Background

In mammals the glottis is protected and closed during swallowing mainly by covering it with the epiglottis, as well as the pulling together (adduction) of the cartilages on either side of the glottis. Birds, however, have a slightly different laryngeal cartilage arrangement to mammals, with both the thyroid and epiglottic cartilages being absent. *Struthio camelus* (ostrich) and *Dromaius novaehollandiae* (emu), in comparison to neognathous birds, possess a very wide glottis [1]. The question can therefore be asked [2] (and remains unanswered) as to how it is possible for these large birds to have such a wide glottis, with apparently little protection, and yet avoid inhalation of food particles and fluid. Despite feeding and drinking studies in *Gallus gallus*[3] and in palaeognaths [4,5] using cinematography and radiography, no attempt has been made to explain or demonstrate how the glottis is protected during swallowing. Unique features necessary to perform this function are noted; however, their role in protecting or covering the glottis is not mentioned.

There appears to be two specifically unique lingual structures associated with *S. camelus* and *D. novaehollandiae*, namely, the pocket in the tongue body of *S. camelus*[2,4,6-11] and the prominent, triangular tongue root of *D. novaehollandiae*[2,12-15]. Despite numerous authors (see above) having noted or described a pocket in the base of the tongue of *S. camelus*, the function of this anatomical peculiarity has remained elusive and only a few authors [4,7,11] have proposed a function for this structure. Similarly, in *D. novaehollandiae*, functions for the tongue root have been suggested [12,13,16] but not conclusively demonstrated.

In this study we aim to marry the functional data on living *Ratidae* and morphological observations on fresh and preserved material to demonstrate how the intricate relationship between the variably structured tongue body, tongue root and laryngeal mound, of *S. camelus* and *D. novaehollandiae*, functions to close off and stabilise the glottis during swallowing, thus partially fulfilling the role of an epiglottis. It is demonstrated that this relationship in the living animal, with such perfectly fitting structures, cannot merely be explained away as a coincidence. This unique proposed anatomical mechanism has tentatively been named the linguo-laryngeal apparatus.

## Results

### *S. camelus*

**Figure 1:**
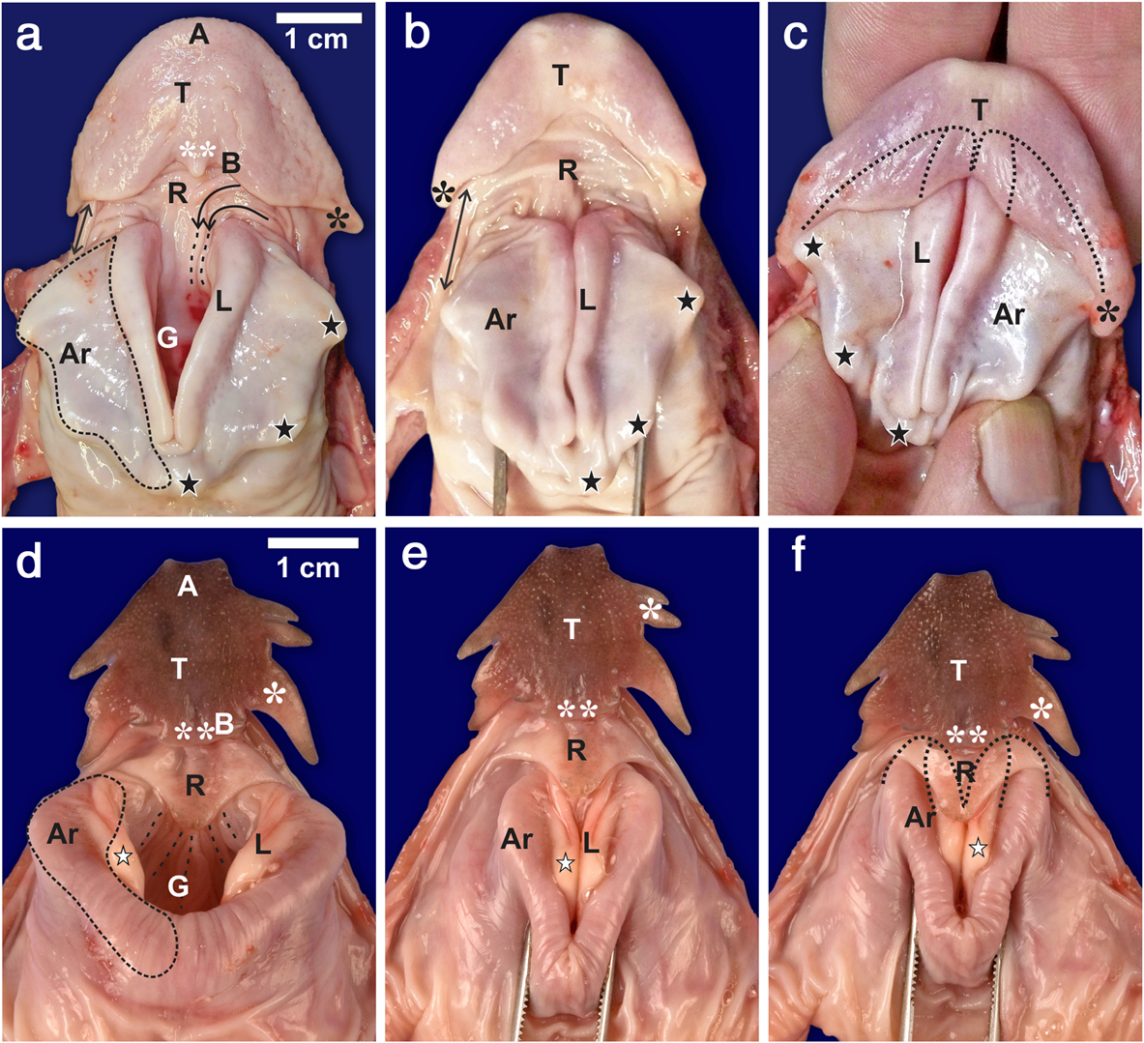
**Sequence of action of the linguo-laryngeal apparatus. a-c.*S. camelus*.** Tongue body (*T*), apex (*A*), tongue body base (*B*), tongue root (*R*), transverse folds (*curved black arrows*) and longitudinal folds (*dotted lines*) of the root, lingual papilla (black *), central lingual papilla (white **), fold between tongue and laryngeal mound (*↔*), glottis (*G*), laryngeal projections (*black stars*), arytenoid cartilage (*dotted outline, Ar*) which also underlies the lips of the glottis (*L*). **a:** Resting position of the tongue and laryngeal mound with an open glottis. **b:** The glottis is in the closed position. **c:** The tongue is retracted and covers the rostral portion of the laryngeal mound and lips of the glottis (*dotted outline*). Note how the lingual papillae hook over the first laryngeal projection. **d-f.*D. novaehollandiae*.** Tongue body (*T*), apex (*A*), tongue body base (*B*), lateral (white *) and caudal (white **) lingual papillae, tongue root (*R*), longitudinal folds (*dotted lines*) on the floor of the larynx, glottis (*G*), lips of the glottis (*L*), protrusion of the lips (*white star*), arytenoid cartilage (*dotted outline, Ar*). **d:** Resting position of the tongue and laryngeal mound with an open glottis. **e:** The glottis is in the closed position. **f:** The tongue is retracted and the tongue root covers the rostral portion of the laryngeal mound and lips of the glottis (*dotted outline*)

**Figure 2:**
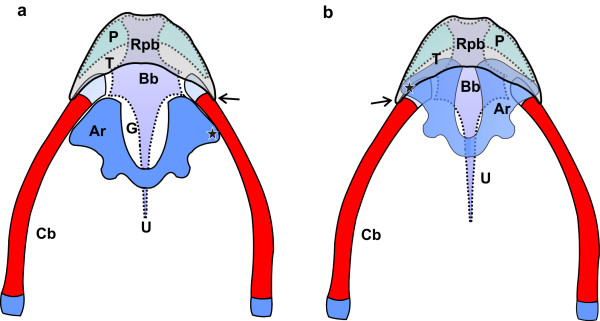
**Schematic representation of the supporting elements of the linguo-laryngeal apparatus of*S. camelus*. Dorsal view.** The cricoid and procricoid cartilages as well as the tongue root and fleshy lips of the glottis are omitted for clarity. The caudal aspect of the arytenoid cartilages (*Ar*) are not joined as indicated but separated by the procricoid cartilage (not illustrated). Red indicates bone and blue/purple cartilage as seen in a 2 week-old chick. **a:** Resting relationship (see Figure 1a) of the arytenoid cartilages, tongue body (*T*), urohyal (*U*), body of the basihyal (*Bb*), rostral projection of the basihyal (*Rpb*), *paraglossalia* (*P*), glottis (*G*), rostral laryngeal projection (*black star*), lingual papilla (*black arrow*) and ceratobranchials (*Cb*). **b:** The relationship of the underlying structures following retraction of the tongue (see Figure 1c). Note how the rostral portions of the arytenoid cartilages are enclosed in the tongue pocket and how the lingual papillae hook over the rostral laryngeal projections. The glottis appears open as the soft tissue (see Figures 1a-c) has not been included in the sketch

**Figure 3:**
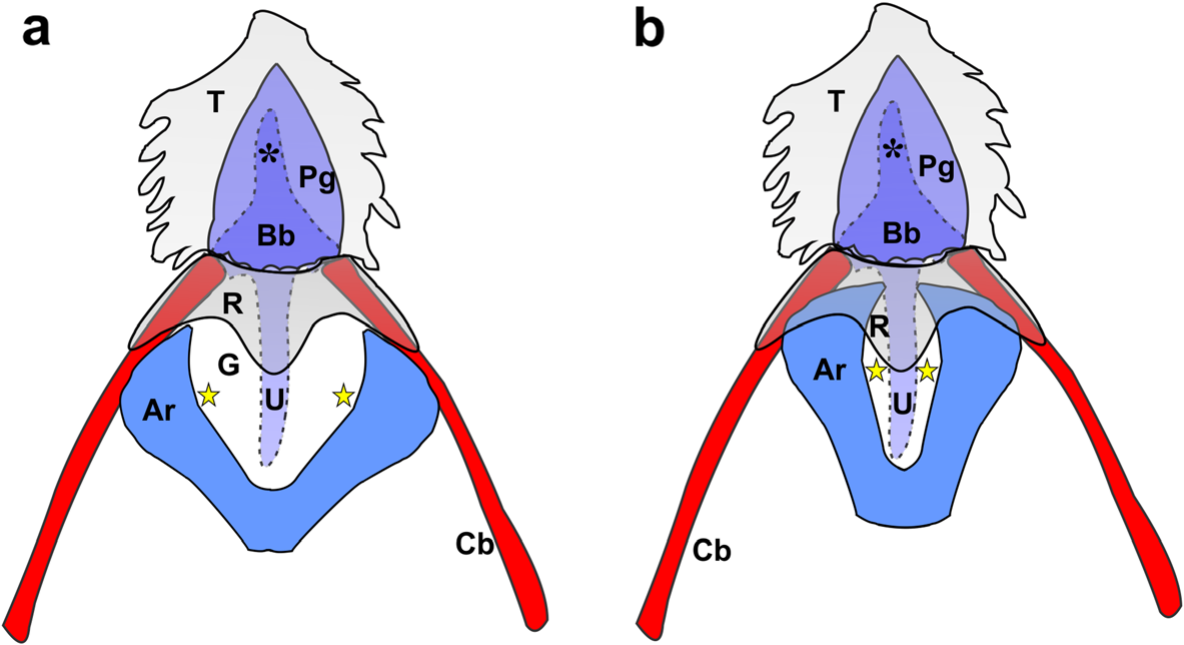
**The lingual pocket of the linguo-laryngeal apparatus of*S. camelus*. a:** The lingual pocket (*caudal margin indicated by arrows*) reflected cranially to reveal a secondary fold (*F*) as well as an additional structure (*dotted outline*) similar in shape to the tongue root of *D. novaehollandiae* (only present in some specimens). Lingual papilla (*), tongue root (*R*), laryngeal projection (*black star*), mucosal covered arytenoid cartilage (*Ar*) and fleshy lips (*L*) of the glottis. **b:** Midline, longitudinal section of the linguo-laryngeal apparatus as seen in Figure 1c. Note how the tongue root concertina’s to allow the rostral portion of the laryngeal mound to enter the lingual pocket (*P*). Tongue body (*Tb*), rostral process of the cricoid cartilage (*Rp*), urohyal (*U*), body of the basihyal (*Bb*) and rostral projection of the basihyal (*Rb*)

Figure 1a depicts the resting relationship of the tongue and laryngeal mound in *S. camelus*. The tongue body was ∩ − shaped with a rounded apex and a concave base (Figures 1a-c). Each lateral margin ended in a small lingual papilla (Figures 1a-c23a), which was attached from its medial aspect by a fold to the laryngeal mound (Figures 1a, b). The smooth margin of the tongue base (Figures 1b, c) displayed a central papilla in some specimens (Figure 1a). The *paraglossum*, unique to *S. camelus*, was in the form of paired cartilaginous *paraglossalia*[4,11] which were situated ventro-laterally in the tongue body (Figure 2). The tongue root was represented by a folded tract of mucosa positioned between the base of the tongue body and the laryngeal mound (Figures 1a, b). Transverse folds converged medially to form longitudinal folds continuous with the floor of the larynx (Figure 1a). The base of the tongue body was hollowed to form a rostrally directed pocket from the floor of which originated a small caudally directed fold of tissue (Figure 3a). In some specimens (Figure 3a) the floor of the pocket, caudal to the small fold, displayed a structure which was similar in shape and orientation to that of the tongue root of *D. novaehollandiae* (see Figures 1d-f4). The raised laryngeal mound was a star-shaped structure with a wide, V-shaped glottis (Figure 1a). The lips forming the glottis were slightly raised above the laryngeal mound, (Figure 3b) contained many mucous glands below the mucosa (personal observation) and were fleshy structures unsupported internally by the underlying arytenoid cartilages. The laryngeal projections were supported by the arytenoid cartilages [11] (Figures 1a-c3a).

**Figure 4:**
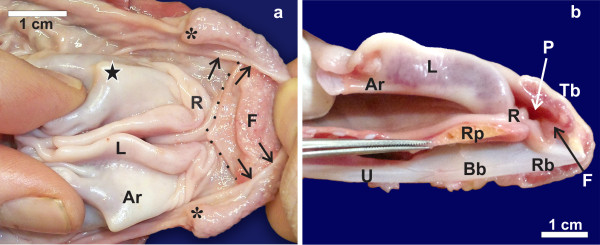
**Rostral view of the linguo-laryngeal apparatus of*D. novaehollandiae*. a:** The normal resting position of the tongue body (*T*), root (*R*) and laryngeal mound. Note the wide glottis (*G*) and the tongue root which protrudes into the glottis. Mucosal covered arytenoid cartilage (*Ar*), lips of the glottis (*L*) and their round protrusion (*star*) and lateral lingual papilla (*). **b:** With the linguo-laryngeal apparatus in position note how the caudal protrusion of the tongue root is positioned over the rostral aspect of the laryngeal mound and adducted glottis and approximates the round protrusions of the glottal lips

During closure (adduction) of the glottis the left and right margins were closely apposed (Figure 1b) but did not appear to completely seal the glottis, particularly the rostral (widest) part (Figure 3a). As the tongue was retracted, the smooth tongue root became highly folded and was effectively obliterated (Figure 3b) as the rostral portion of the laryngeal mound was encased by the pocket in the base of the tongue body (Figures 1c2b). Concurrently, the lingual papillae hooked over the most rostral laryngeal projections (Figures 1c2b). In this fashion the lingual pocket and lingual papillae effectively encapsulated and stabilised the rostral portion of the laryngeal mound (Figures 1c2b), which was the weakest point of the adducted glottis. The relatively mobile, paired cartilaginous *paraglossalia* situated within the tongue body (Figure 2) allowed a measure of flexibility and sufficient rigidity to the organ, facilitating its action. The elements of the hyobranchial apparatus have been described [4,11], and as seen in a median longitudinal section, the urohyal, body and rostral projection of the basihyal together with the arytenoids and rostral projection of the cricoid cartilage, appeared to form a firm base onto which the lingual pocket could ‘clamp’ to secure the laryngeal mound and adducted glottis (Figure 3b).

### *D. novaehollandiae*

Figures 1d and 3a depict the resting relationship between the tongue body, root and laryngeal mound of *D. novaehollandiae*. These structures have previously been described in detail [12,14]. In summary, the tongue body was triangular and the lateral margins were adorned with numerous lingual papillae which varied in shape and number (Figures 1d-f3). The base was rounded caudally due to the presence of one or more caudal papillae. The prominent tongue root was triangular with a round, raised, caudally directed protrusion advancing a short distance into the glottis (Figures 1d-f3). Caudal to the tongue root, on the floor of the larynx, were three to five longitudinal mucosal folds (Figure 1d). The laryngeal mound was rhomboid-shaped and the glottis was widened rostrally, slightly convex medially and narrowed at the caudal end (Figures 1d3a). As in *S. camelus*, the lips of the glottis were formed by a mucosa, unsupported internally by the underlying arytenoid cartilages. The lips of the glottis sloped dorso-caudally and at the highest point displayed a small, round protrusion (Figures 1d-f3).

**Figure 5:**
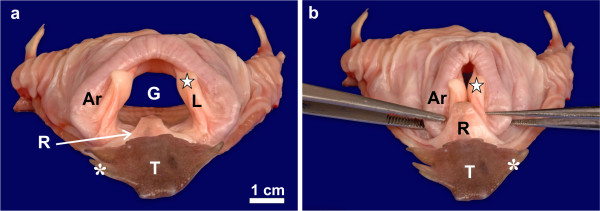
**Schematic representation of the supporting elements of the linguo-laryngeal apparatus of*D. novaehollandiae.*Dorsal view.** The cricoid and procricoid cartilages as well as the fleshy lips of the glottis are omitted for clarity. The caudal aspect of the arytenoid cartilages (*Ar*) are not joined as indicated but separated by the procricoid cartilage (not illustrated). However, the relative position of the protrusion of the fleshy lips (*yellow star*) (see Figures 1d-f, 4) have been indicated. Red indicates bone and blue/purple cartilage as seen in an 8 week-old chick. **a:** Resting relationship (see Figure 1d) of the arytenoid cartilages, tongue body (*T*), tongue root (*R*), urohyal (*U*), body of the basihyal (*Bb*), rostral projection of the basihyal (*), *paraglossum* (*Pg*), glottis (*G*) and ceratobranchials (*Cb*). **b:** The relationship of the underlying structures following retraction of the tongue (see Figure 1f). Note how the rostral portions of the arytenoid cartilages are enclosed by the tongue root and how the protrusions of the lips of the glottis and the tongue root close off the glottis

During closure of the glottis (Figure 1e) a small gap was noticeable at the rostral aspect due to the slight concavity of the lips of the glottis. As the tongue was retracted, the tongue root moved caudally together with the tongue body and slid over the rostral portion of the laryngeal mound and adducted glottis (Figures 1f4b5b). In this way the rounded caudal protrusion of the tongue root met with the round protrusions of the lips of the glottis (Figures 1f4b5b), and effectively closed the small gap in the adducted glottis (Figures 1f). Simultaneously, the rostral portion of the laryngeal mound and weakest point of the adducted glottis was enclosed and stabilised by the tongue root and the base of the tongue body, which was stiffened by the presence of the cartilaginous *paraglossum*[12] (Figure 5). A shallow recess in the tongue base [12] allowed the tongue body to slide a short distance caudally over the tongue root, thus stabilising its position.

## Discussion

Until recently the only reported action of the ratite tongue during feeding was retraction during swallowing [4] and depression of the mouth floor to allow for the effective intra-oral transfer of food to the proximal oesophagus using the ‘catch and throw’ method [5]. The ratite tongue has been classified as rudimentary and of little functional significance during feeding when compared to the tongues of neognaths [5]. However, recent morphological studies have revealed a number of diverse functions for this organ in ratites [12,13,17]. These include swallowing, cleaning of the palate [12], lubrication, mechanoreception, taste and mechanical and immune protection [13] in *D. novaehollandiae*, and cleaning of the choana in *R. americana*[17]. Furthermore, as demonstrated here, the ratite tongue potentially plays a far more pivotal role during feeding by closing off the weakest part of the adducted glottis during swallowing and thus preventing the inhalation of food and water (choking). This appears to be achieved, in *S. camelus*, by folding of the flat tongue root which allows the lingual pocket to encase the adducted glottis, and in *D. novaehollandiae*, by the specialised structure of the tongue root which slides over the rostral aspect of the adducted glottis. We have termed this proposed mechanism the linguo-laryngeal apparatus.

Despite the lingual pocket in *S. camelus*[2,4,6,8-11] and triangular tongue root in *D. novaehollandiae*[2,12] having been described, why has such an elegant mechanism eluded discovery for so long? Possible explanations for the prolonged obscurity of this mechanism are, firstly, most morphological studies are conducted on preserved material. Regardless of the fixative used (commonly formalin or alcohol) the tissues become hardened and the manipulations performed here on fresh specimens are impossible in preserved tissues. Secondly, functional studies [4,5] using diagnostic imaging techniques do not detect the soft tissue and cartilage adequately or provide an intra-oral view thus making it impossible to demonstrate or interpret the movements we have described. Additionally, and only compounding the problem, it was generally accepted that the small ratite tongue was rudimentary, and therefore of little functional significance.

The proposed functioning of the linguo-laryngeal apparatus would rely on muscle action decreasing the distance between the hyobranchial apparatus (and thus tongue) and the larynx. The detailed study on the musculature of the hyobranchial apparatus of *S. camelus* and *D. novaehollandiae*[4] provides supportive evidence in this regard. The muscle groups responsible for decreasing the distance between the two components are the hyolaryngeal and extrinsic hyolingual retractor muscles. The *M. cricohyoideus*, a hyolaryngeal muscle, appears to be a powerful muscle in *S. camelus* and is partially located in the tongue body which is a situation unknown in other taxa [4]. The *M. ceratocricoideus* is unique to paleaognaths and is considered an unusual hyolaryngeal muscle [4]. The hyolaryngeal muscles contract to decrease the distance between the basiurohyal (and thus the tongue) and the larynx during retraction of the tongue [4]. This muscular action would account for the proposed functioning of the linguo-laryngeal apparatus. Additionally, the extrinsic hyolingual retractors also show some unique features in ratite species. In *S. camelus* the *M. serpihyoideus* inserts directly on the cricoid cartilage, again a situation unique in known avian taxa [4]. However, in *D. novaehollandiae* this muscle acts directly on the ceratobranchials and urohyal [4]. The *M. hyomandibularis* acts directly on the ceratobranchials in *S. camelus* but in *D. novaehollandiae* shows a unique configuration not reported in other avian taxa and is divided into an *M. hyomandibularis lateralis* (inserting on the mid-ceratobranchial) and *M. hyomandibularis medialis* (inserting on the urohyal) [4].

In *S. camelus* it would appear that the main muscle pulling the tongue, and thus the lingual pocket, over the adducted glottis would be the *M. cricohyoideus*, which in *S. camelus* has a unique conformation. In *D. novaehollandiae* the main contributors to pulling the tongue root over the adducted glottis would appear to be those muscles attaching to the urohyal, namely the *M. serpihyoideus* and *M. hyomandibularis medialis* of which the latter is again unique to *D. novaehollandiae*. The existence, conformation and positioning of such unique muscles, coupled with the synergy of the precisely formed anatomical structures reported in this study, further support the proposed functioning of the linguo-laryngeal apparatus.

There is currently no consensus as to the presence of a structure that functionally replaces the epiglottis in birds. It was originally suggested [7] that the posterior border of the tongue of *S. camelus* functioned like an epiglottis; however, this was later refuted [2]. To further support this observation [7] in *S. camelus*, as well as our own conclusions, it was noted that a fold in the base of the tongue of *Apteryx australis* covers the glottis when the tongue is retracted [18]. Other suggestions as to the function of the lingual pocket in *S. camelus* add further support to our findings on the linguo-laryngeal apparatus. The lingual pocket, observed by high-speed cineradiography, is reported to change shape during intraoral transport of food and is closed during tongue retraction [4]. However, the pocket may have appeared ‘closed’ due to it being filled by the rostral aspect of the laryngeal mound as the tongue is pulled caudally (see Figures 1c2b3b). The muscular action of the *M. ceratoglossus* on the *paraglossalia*, ‘closing’ the pocket during swallowing [4], would undoubtedly aid in anchoring the pocket over the rostral aspect of the laryngeal mound. A secondary fold is present in the lingual pocket, which would provide an increase in surface area for mucus-producing glands, enhancing mucus production and secretion for the ingestion of dry food [11]. This additional mucus would facilitate a smooth sliding motion of the lingual pocket over the laryngeal mound. In *D. novaehollandiae* it was originally proposed that the tongue root functioned like an epiglottis [16] but, as in *S. camelus*, this was subsequently refuted [2]. However, this role for the tongue root of *D. novaehollandiae* was again proposed [12] and has now been tentatively demonstrated. It may be possible that neognathous birds (the jay and flamingo [8] and the chicken and domestic birds [19]) possess similar mechanisms (although less specialised), as has been previously suggested. This mechanism consists of a transverse, semi-lunar fold at the entrance to the glottis [8] or folds opposite the base of the tongue [19] that can function as a rudimentary epiglottis. However, the action of these folds was never functionally demonstrated which is most likely why their proposed role as an “epiglottis” has not been accepted and recognised. In the chicken, this mechanism does seem possible where, in the fresh state, the flat, smooth tongue root (which is relatively long) forms a semi-circular fold when the tongue is retracted and which covers the rostral part of the glottis (personal observation). Thus it has been suggested and debated, but never conclusively stated, that the tongue root in birds may function, albeit partially, as a form of epiglottis.

## Conclusions

This study finally proposes a more explicit function of the peculiar lingual structures reported in *S. camelus* and *D. novaehollandiae* during the past 177 years and may explain why these intriguing birds do not choke. In the absence of an epiglottis, the wide glottis of ratites appears to be protected by the linguo-laryngeal apparatus (which varies in structure between ratite species studied to date) and which may functionally replace the epiglottis. Although the muscles acting on the tongue of *S. camelus* and *D. novaehollandiae* have been described [4], further studies on these muscles, in light of this newly described mechanism, could further elaborate, confirm and explain this action. Based on our initial findings on how the glottis is protected in *S. camelus* and *D. novaehollandiae* we propose that a functional mechanism which protects the glottis does exist in birds. Whereas mammals possess an epiglottis, ratites (and possibly birds in general), possess a multi-component mechanism, the linguo-laryngeal apparatus. The linguo-laryngeal apparatus functions through the synergy created by a number of specialised anatomical components (the tongue body, tongue root, supporting elements of the tongue (bones and cartilage), laryngeal mound and its supporting cartilages). Furthermore, the linguo-laryngeal apparatus represents a newly discovered functional mechanism which should be incorporated into the field of avian biology.

## Methods

We collected the heads from five adult *S. camelus* and five adult *D. novaehollandiae* of either sex that had been slaughtered at a commercial abattoir. The tongues and laryngeal mounds were removed by cutting through the arms of the ceratobranchials and frenulum and freeing the structures from the oropharyngeal floor. The fresh specimens were washed in running tap water to remove blood and debris. Additionally, tongues with attached laryngeal mounds from one 2 week-old *S. camelus* chick and one 8 week-old *D. novaehollandiae* chick, which were part of the departmental collection, were stained for cartilage (alcian blue) and bone (alizarin red), and the tissues cleared [20] to facilitate a description of the internal supporting elements of the tongue and laryngeal mound. As the specimens were fixed in formalin for more than 2 hours they were first rinsed in running tap water for 24 hours prior to staining [20]. We performed appropriate manipulations on the specimens aimed at mimicking the postulated movements that occur during swallowing. These manipulations are not possible in formalin-fixed specimens as fixation does not allow for free movement of the structures involved. Manipulations were also possible in the two stained specimens as the tissues were treated with Trypsin and were rendered soft and moveable. Adduction (closure) of the glottis was achieved by using forceps (Figures 1b, e, f) or fingers (Figures 1c3a) to apply pressure at the base of the arytenoid cartilages. The tongue body was moved caudally by digital manipulation or by using forceps. The sequence of movements and interactions was observed for each specimen, recorded digitally using a Canon 5D digital camera equipped with a Canon Macro 100 mm lens, and described. The specimens were subsequently stored in 10% neutral-buffered formalin and the stained specimens in glycerol. The terminology used in this study is that of Nomina Anatomica Avium [21].

## Misc

Martina R Crole and John T Soley contributed equally to this work

## Competing interests

The authors declare that they have no competing interests.

## Author’s contributions

MRC took the primary lead in most aspects of the work and compilation of the paper. The concept was the original idea of MRC and was supported and built upon by JTS. JTS acted in a supervisory role on all aspects of the work and was responsible for the refinement of the manuscript. Both authors collected the specimens, discussed the results and contributed equally to the manuscript. All authors read and approved the final manuscript.

## Author’s details

MRC is a Senior Lecturer and PhD student in the Department of Anatomy and Physiology, Faculty of Veterinary Science, University of Pretoria. MRC’s research focus is on the detailed comparative anatomy of the oropharynx of ratite species. JTS is a Professor in the same department as MRC and has 25 years of experience working on various aspects of ratite anatomy. The research of MRC and JTS comprises detailed descriptive studies aimed at elucidating underlying functions.
